# Genomic epidemiology of human parvovirus B19 in Southern Brazil, 2015–2024

**DOI:** 10.1007/s11033-026-13080-3

**Published:** 2026-10-30

**Authors:** Tatiana Schaffer Gregianini, Thales de Lima Bermann, Érica Möllman, Fernanda Martiny, Helena Costa Munhós, Amanda Pellenz Ruivo, Milena Bauermann, Lara Crescente, Juliana Patzer, Ludmila Fiorenzano Baethgen, Zenaida Nunes, Gabriel da Luz Wallau, Ana Beatriz Gorini Veiga, Richard Steiner Salvato

**Affiliations:** 1 Laboratório Central de Saúde Pública, Centro Estadual de Vigilância em Saúde, Secretaria Estadual da Saúde do Rio Grande do Sul, Porto Alegre, Brazil; 2 Departamento de Genética, Instituto de Biociências, Programa de Pós-Graduação em Genética e Biologia Molecular (PPGBM), Universidade Federal do Rio Grande do Sul, Porto Alegre, Brasil; 3 Universidade Federal de Ciências da Saúde de Porto Alegre, Porto Alegre, CEP: 90050-170 Brazil; 4 Divisão de Vigilância Epidemiológica, Centro Estadual de Vigilância em Saúde, Secretaria Estadual da Saúde do Rio Grande do Sul, Porto Alegre, Brazil; 5 Instituto Aggeu Magalhães, Fundação Oswaldo Cruz, Recife, Brazil; 6 Department of Arbovirology and Entomology, Bernhard Nocht Institute for Tropical Medicine, Hamburg, Germany; 7 Universidade Federal de Santa Maria, Santa Maria, Brazil; 8 Departamento de Análises Clínicas, Bromatológicas e Toxicológicas, Faculdade de Ciências Farmacêuticas de Ribeirão Preto, Universidade de São Paulo, Ribeirão Preto, Brazil

**Keywords:** Parvovirus B19, Molecular epidemiology, Erythema infectiosum

## Abstract

**Background:**

Human parvovirus B19 (B19V) causes erythema infectiosum and, in severe cases, has been associated with hydrops fetalis and transient aplastic crises. Despite its clinical importance, particularly in vulnerable groups such as pregnant women and immunocompromised individuals, epidemiological and genomic data on B19V remain limited.

**Methods and Results:**

We investigated 4,118 samples collected from patients with exanthematous illness in Rio Grande do Sul, Southern Brazil, between 2015 and 2024 using serological and molecular testing, and developed an amplicon-based whole-genome sequencing protocol for B19V, generating 47 near-complete genomes. B19V was detected across all age groups, with a higher frequency among females (62.4%), and among adults aged 21–40 years. Viral circulation was detected year-round, with a modest increase in July and a more pronounced peak in September, consistent with Southern Hemisphere winter seasonality. Forty-one genomes from the 46 included in the dated phylogeny clustered in a single well-supported clade containing sequences from other Brazilian states, with Rio Grande do Sul sequences interspersed rather than forming a single lineage derived from one introduction. The remaining five genomes clustered with sequences from other regions of Brazil and Argentina. This phylogenetic pattern reflects sustained endemic circulation through repeated local transmission and is not adequately captured by current genotype-level classification systems.

**Conclusions:**

The findings of this study expand the genomic understanding of B19V in South America and provide important insights into its molecular epidemiology, seasonal patterns, and evolutionary history in Southern Brazil.

**Supplementary Information:**

The online version contains supplementary material available at https://doi.org/10.1007/s11033-026-13080-3.

## Introduction

Human parvovirus B19 (B19V) is a small non-enveloped single-stranded DNA virus that belongs to the *Parvoviridae* family, subfamily *Parvovirinae* [1]. B19V infection may cause exanthematous illness such as erythema infectiosum, and, in severe cases, it has been associated with hydrops fetalis and erythroid aplasia. The main B19V route of transmission is through respiratory secretions; however, the virus can also be transmitted parenterally and through transplacental (vertical) transmission [2].

Following the COVID-19 pandemic, the incidence of B19V infections has surged globally, particularly among younger populations. Recent surveillance data from the United States and widespread outbreaks across Europe since early 2024 support this resurgence [3, 4]. Most infections are asymptomatic or mild and self-limiting. In children, B19V typically presents as erythema infectiosum with mild flu-like symptoms. In adults, it more often causes acute arthralgia or arthritis, especially in women [5]. However, in high-risk groups, including immunocompromised individuals, pregnant women, and those with hematologic disorders, B19V infection can lead to severe complications. These include transient aplastic crisis in individuals with hemolytic disorders, chronic anemia in immunocompromised patients, and hydrops fetalis following maternal infection, which may result in fetal death [6, 7].

B19V is classified into three distinct genotypes and shows significant genetic diversity, which may affect its epidemiology, pathogenicity, and evolution [8–10]. Genotype 1 is globally prevalent, genotype 2 is primarily found in the tissues of elderly individuals but is only sporadically detected as a circulating virus, and genotype 3 is restricted to specific geographical regions, such as Western Africa. Notably, the latter shows greater genetic variability than genotypes 1 and 2, which may reflect longer-term circulation or a possible ancestral origin in this region [11]. In Brazil, laboratory diagnosis of B19V is primarily performed as part of the differential diagnosis of febrile exanthematous diseases, particularly in samples that test negative for measles and rubella, which are notifiable exanthematous diseases. This diagnostic workflow typically includes human herpesvirus 6, dengue, chikungunya, Zika virus, and B19V. The selection of pathogens to be tested in the diagnostic routine is guided by the local epidemiological context, including ongoing outbreaks, vaccination coverage, and inconclusive initial results. Brazilian public health laboratories usually use Enzyme-Linked Immunosorbent Assay (ELISA) to diagnose acute B19V infection by detecting IgM antibodies from the first days through approximately four weeks after rash onset [12].

Despite its clinical relevance, including severe clinical implications for maternal-fetal and hematological health, B19V is not a mandatory notifiable pathogen in Brazil, resulting in profound structural underreporting. Additionally, because acute B19V infections frequently resemble arboviral diseases, many cases remain undetected or are incorrectly diagnosed. Thus, data on the epidemiology and molecular characteristics of B19V in Brazil remain limited, largely restricted to case reports, outbreak investigations, and small population studies, with most evidence coming from the Southeast region. A recent retrospective analysis of B19V in Brazil identified genotype 1 as the predominant circulating genotype country-wide and highlighted the virus as a hidden and neglected pathogen, often masked by arboviral infections such as dengue, Zika, and chikungunya. Notably, data from Rio Grande do Sul (RS), the southernmost state in Brazil, are particularly scarce, with only a single documented B19V case in the state, recorded in 2018 [13].

Understanding B19V genetic diversity in southern Brazil is important for developing effective diagnostic tools and guiding better surveillance strategies as well as infection control and treatment. To address this gap, we conducted a molecular epidemiology study on B19V strains circulating in Rio Grande do Sul over a ten-year period. Our findings demonstrate sustained B19V circulation in Rio Grande do Sul, characterized by recurrent seasonal activity and a predominantly interconnected viral population rather than repeated independent introductions, providing new insights into the B19V local transmission dynamics and evolutionary history.

## Materials and methods

### Sample collection and ethics aspects

Between 2015 and 2024, the Rio Grande do Sul State Public Health Laboratory (LACEN-RS) received serum samples from patients suspected of measles or rubella. Blood samples were collected from the first day of rash onset up to 30 days after symptom onset (preferably before the 5th day) and sent to LACEN-RS in serum-separation tubes. After ruling out measles or rubella, we screened the samples for parvovirus B19 (B19V) using enzyme-linked immunosorbent assay (ELISA). Among serologically positive specimens, those collected early after symptom onset were further analyzed by real-time PCR to detect B19V. The Ethics Committees of the Escola de Saúde Pública do Rio Grande do Sul (CAAE 67181123.1.0000.5312) and the Universidade Federal de Ciências da Saúde de Porto Alegre (CAAE 75118217.9.0000.5345) approved the study. The requirement for informed consent was waived, as samples were originally obtained for routine public health surveillance purposes.

### Serological testing

We centrifuged the received samples without anticoagulant to obtain serum and stored them at 4 °C to 8 °C. ELISA for B19V antibody detection was performed using the following commercial kits: Vircell Parvovirus ELISA (IgM) (Granada, Spain), Euroimmun Parvovirus B19 (IgM) (São Paulo, Brazil), and SERION ELISA classic Parvovirus B19 IgM (Würzburg, Germany). We used all kits according to the manufacturer’s instructions. We used each kit during different periods throughout the study, depending on supply availability.

### Molecular testing

We isolated viral deoxyribonucleic acid (DNA) from samples collected within up to five days of symptom onset using the Extracta 96 automated extractor (Loccus) with the Fast Viral DNA and RNA kit (MVXA-P096 FAST) (Loccus, São Paulo, Brazil), following the manufacturer’s instructions. For molecular diagnosis and to identify samples during the viremic phase (suitable for subsequent genomic sequencing), we detected B19V by real-time polymerase chain reaction (PCR) using a previously published methodology [14]. Samples that showed amplification with a cycle threshold (Ct) < 40 were considered positive for B19V. We performed reactions on a CFX Opus Real-Time PCR System (BIO-RAD, Hercules, United States).

### B19V genomic sequencing

We randomly selected five samples (with Ct values ranging from 15 to 28 for B19V in real-time PCR) for metagenomic sequencing using a hybrid–capture-based approach with the Illumina Viral Surveillance Panel (Illumina, San Diego, USA), following the manufacturer’s instructions. We assembled the resulting FASTQ reads in Illumina BaseSpace using the *de novo* assembly tool in the DRAGEN Microbial Enrichment Plus application. Using this approach, we recovered the complete B19V sequence from all five samples. We then combined the assembled genomes with the reference genome NC_000883.2 and used them as input for varVAMP [15] to design a primer scheme to generate overlapping amplicons covering the entire B19V genome (Supplementary Table 1). The resulting alignment used as input for varVAMP, as well as sequencing quality metrics for generated sequences, are available at https://github.com/salvatolab/B19V-genomic-epidemiology-Rio-Grande-do-Sul.

We then conducted an in silico validation of the designed primer scheme. First, we downloaded all 503 B19V sequences with at least 70% genome coverage available in the NCBI GenBank database (https://www.ncbi.nlm.nih.gov/) as of September 2026. We aligned these sequences using MAFFT [15]. We then used an in-house Python script to assess primer-binding-site conservation and systematically predict potential genomic dropouts.

Next, we used the primer scheme designed by varVAMP to amplify complete genome sequences from an additional 50 samples that showed a Ct ≤ 28 in real-time PCR. Each PCR reaction (25 µL total volume) contained: 2.5 µL of 10× PCR buffer, 0.5 µL of 10 mM dNTP mix, 0.8 µL of 50 mM MgCl₂, 2 µL of B19V primer pool, 0.2 µL of Platinum™ Taq DNA polymerase (Invitrogen, USA), and 16.5 µL of nuclease-free water, plus 2.5 µL of extracted DNA template. PCR cycling conditions were as follows: initial denaturation at 98 °C for 30 s; 40 cycles of 98 °C for 15 s and 65 °C for 5 min; followed by a final hold at 4 °C. We checked the resulting amplicons by gel electrophoresis. We then constructed genomic libraries using the Illumina DNA Prep kit (Illumina, San Diego, USA), according to the manufacturer’s instructions. We sequenced samples on the Illumina MiSeq platform using the V3 600-cycle kit, generating paired-end reads (2 × 150 bp).

### Genome assembly and phylogenetic analysis

Reference-based genome assembly was performed using the ViralFlow 1.0 pipeline [29], with the closely related and well-annotated B19V reference genome (GenBank accession number: NC_000883.2). After quality control, we excluded eight sequences with genome coverage below 70%, leaving a final dataset of 47 newly generated B19V genome sequences. To characterize B19V phylogenetic patterns, we downloaded all publicly available near-complete B19V genome sequences with genome coverage greater than 70% from the NCBI database in September, 2026. After curation, we retained 446 publicly available genomes from 26 countries, collected between 2002 and 2025. The 446 curated genomes were aligned with the 47 sequences generated in this study, the genotype 1 reference genome (NC_000883.2), and four additional reference strains representing genotypes 2 (LaLi and A6) and 3 (D91.1 and V9), using MAFFT [15]. We manually curated the resulting alignment in AliView [30] to remove alignment artifacts. We reconstructed maximum-likelihood (ML) phylogenetic trees using IQ-TREE 2 [31] under the TIM3 + F+R4 nucleotide substitution model, selected as the best-fitting model by ModelFinder [32], with 1,000 ultrafast bootstrap replicates. We assigned genotypes based on each sequence’s phylogenetic placement relative to the reference strains.

### Phylodynamic analysis

We performed time-resolved phylogenetic analysis using the 443 genotype 1 genomes identified in the ML tree. Of these, 423 had interpretable collection dates, including 47 genomes generated in this study and 376 publicly available genomes, 34 of which originated from other Brazilian states. We assessed the temporal signal by regressing root-to-tip genetic divergence against collection dates using an ML tree rooted by least-squares regression. We excluded genomes with residuals exceeding four interquartile ranges (11 publicly available genomes and one genome generated in this study), resulting in a final dataset of 411 genomes collected between 2002 and 2025. We inferred time-calibrated phylogenetic trees using the Nextstrain Augur toolkit [33] and TreeTime [34]. We reconstructed an ML tree for this subset using IQ-TREE 2 under the TIM3 + F + I+R3 nucleotide substitution model, selected as the best-fitting model by ModelFinder, with 1,000 ultrafast bootstrap replicates. We rooted the tree using least-squares regression against collection dates and calibrated it under a strict molecular clock model, with marginal date inference and a skyline coalescent prior. Ancestral geographic states were reconstructed at the country and continent levels using *mugration* model, treating geographic locations as discrete traits and inferring their most likely states at internal nodes along the time-scaled phylogeny.

### Statistical analysis

We tabulated epidemiological data in Microsoft Excel and analyzed them in Python 3.13 with SciPy 1.17. We reported proportions with 95% confidence intervals calculated using the Wilson method, and Ct values as median, mean, standard deviation, and range. We evaluated the correlation between patient age and Ct values using Pearson’s and Spearman’s rank correlation coefficients. We compared the proportion of female cases with parity using a two-sided exact binomial test. We compared B19V positivity across years and the distribution of sex across years using chi-square tests of independence, recalculating p-values by Monte Carlo resampling with 20,000 replicates when expected counts fell below five. All tests were two-sided, and *p* < 0.05 was considered significant.

We selected nucleotide substitution models using the Bayesian information criterion in ModelFinder, and estimated branch support from 1,000 ultrafast bootstrap replicates, with values of 90 or above considered well supported. We regressed root-to-tip divergence on collection date using ordinary least squares, and excluded tips with residuals beyond four interquartile ranges from the time-resolved analysis. Node ages are reported with the 95% confidence intervals of the marginal date inference in TreeTime, and the clock rate with its standard deviation. Phylogenetic and phylodynamic analyses used MAFFT v7.526, AliView v1.31, IQ-TREE 2, Augur v33.1.0, and TreeTime v0.11.5.

## Results

### Epidemiology of B19V in Rio Grande do Sul, Brazil

**Fig. 1:**
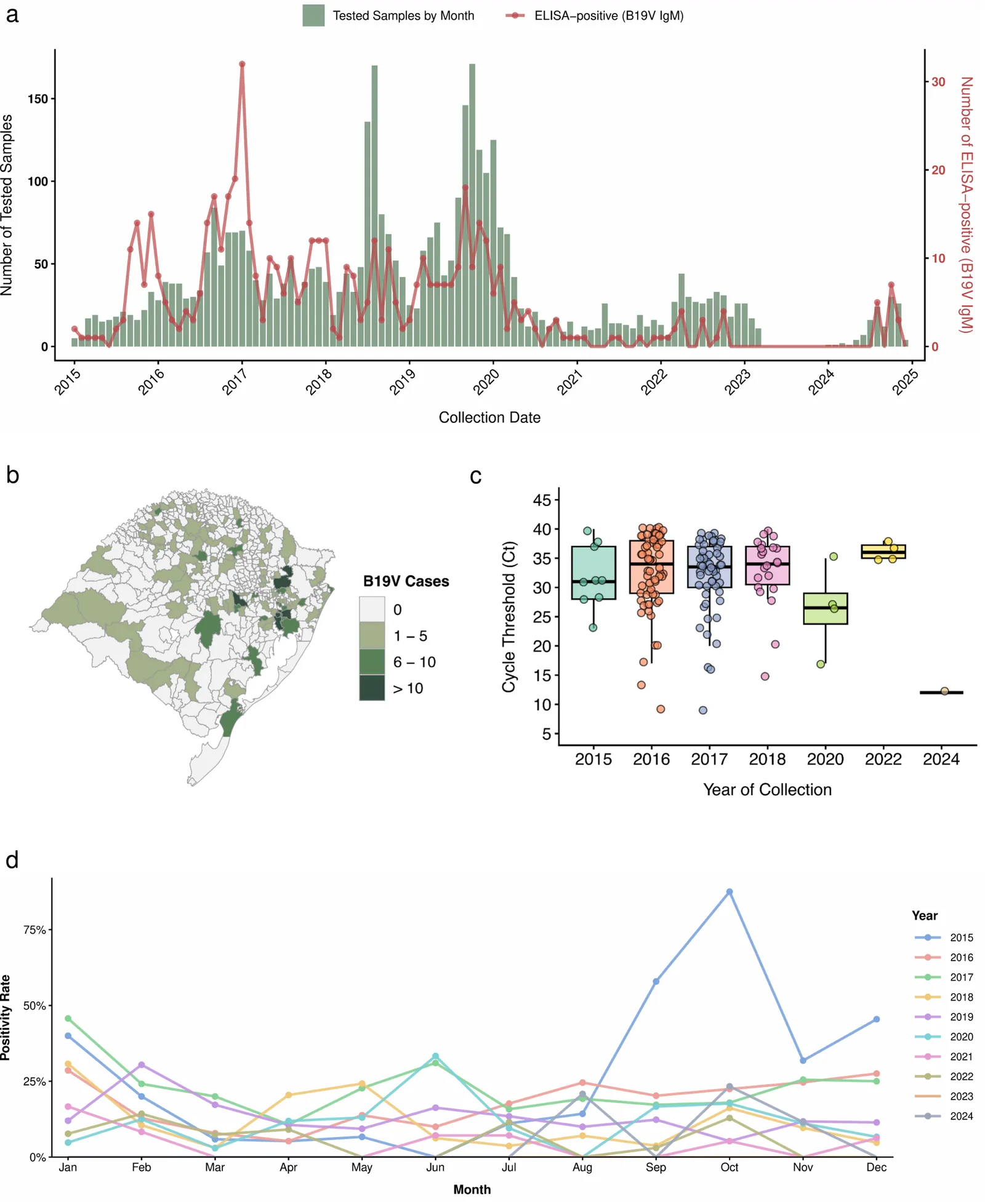
Spatiotemporal distribution and seasonality of parvovirus B19-positive samples detected in Rio Grande do Sul, 2015–2024. **(a)** Total number of samples tested for B19V throughout the period (2015–2025) (green bars) and the number of samples that tested positive for B19V by ELISA (red line). **(b)** Map of Rio Grande do Sul showing the cumulative number of ELISA-positive B19V cases per municipality over the ten-year study period. Municipalities are color-coded by total positive samples in the period. **(c)** Distribution of cycle threshold (Ct) values from real-time PCR across the 160 PCR-positive samples. Each dot represents one sample, and dots are color-coded by year of collection. **(d)** Monthly positivity rate for ELISA-positive B19V cases, with one line per year of the study period

Between 2015 and 2024, a total of 4,118 serum samples from patients suspected of exanthematous diseases that tested negative for measles and rubella were tested for B19V in Rio Grande do Sul, Brazil. Of these, 553 tested positive for B19V by ELISA, for an overall positivity of 13.4% (95% CI 12.4–14.5) (Fig. 1a). Positivity varied across years, from 28.2% (95% CI 22.5–34.7) in 2015 to 3.7% (95% CI 1.7–7.8) in 2021, with no positive result among the 54 samples tested in 2023 (χ² = 172.7, df = 9, *p* < 0.001). Among the 553 B19V-positive cases, 345 (62.4%) were female. However, in the total tested population with available demographic data, overall positivity was 14.7% (95% CI: 13.4–16.2%) for females (345/2,341) and 14.6% (95% CI: 12.9–16.6%) for males (208/1,422). These sex-specific positivity rates were consistent across the study period. Infections were identified across all age groups, with the highest proportion of total positive cases occurring in individuals aged 21–40 years (35.3%; 95% CI 31.4–39.3). However, when adjusting for the total tested population to determine age-specific positivity rates, the highest positivity rate occurred among school-aged children (6–10 years) at 22.3% (95% CI 17.8–27.6). In contrast, the positivity rates for the 21–30 and 31–40 age groups were 13.1% (95% CI 10.9–15.7) and 16.8% (95% CI 13.9–20.1), respectively, while the lowest detection rates were observed in infants under 6 months (4.4%; 95% CI 3.0–6.4) and individuals over 60 years (3.8%; 95% CI 1.0–12.8) (Table 1).

**Table 1:** Characteristics of positive B19V cases from 2015 to 2024 in Rio Grande do Sul, Brazil

Variables	2015	2016	2017	2018	2019	2020	2021	2022	2023	2024	Total
Tested samples	206	564	522	764	973	432	164	311	54	128	4118
B19V ELISA-positive	58	109	128	73	110	38	6	16	0	15	553
PCR-positive	9	64	56	22	0	4	0	4	0	1	160
Positivity rates (95% CI*)	28.2 (22.5–34.7)	18.8 (15.7–22.2)	24.9 (21.4–28.8)	9.7 (7.8–12.0)	11.3 (9.5–13.4)	8.8 (6.5–11.8)	3.7 (1.7–7.8)	5.1 (3.2–8.2)	0.0 (0.0–6.6)	11.7 (7.2–18.4)	13.4 (12.4–14.5)
Sequenced genomes	4	19	13	4	0	3	0	4	0	0	47
Sex											
Female (%)	37 (63.7)	70 (64.2)	93 (72.7)	43 (58.9)	60 (54.5)	22 (57.9)	3 (50.0)	8 (50.0)	0	9 (60.0)	345 (62.4)
Positivity rates (95% CI*)	31.9% (24.1–40.8)	19.4% (15.7–23.8)	28.6% (24.0–33.8)	10.6% (8.0–14.0)	11.1% (8.7–14.0)	9.5% (6.4–14.0)	3.4% (1.2–9.5)	4.7% (2.4–9.0)	0.0% (0.0–11.0)	12.3% (6.6–21.8)	14.7% (13.4–16.2)
Male (%)	21 (36.2)	39 (35.8)	35 (27.3)	30 (41.1)	50 (45.5)	16 (42.1)	3 (50.0)	8 (50.0)	0	6 (40.0)	208 (37.6)
Positivity rates (95% CI*)	24.4% (16.6–34.5)	29.1% (22.1–37.3)	26.7% (19.9–34.9)	9.4% (6.7–13.1)	13.2% (10.2–17.0)	9.7% (6.1–15.2)	6.2% (2.1–16.8)	7.5% (3.9–14.2)	0.0% (0.0–22.8)	14.3% (6.7–27.8)	14.6% (12.9–16.6)
Age											
0–6 m (%)	0	5 (4.6)	1 (0.8)	2 (2.7)	10 (9.1)	4 (10.5)	0	2 (12.5)	0	2 (13.3)	26 (4.7)
Positivity rates (95% CI*)	0.0% (0.0–27.8)	4.5% (1.9–10.1)	1.2% (0.2–6.3)	2.2% (0.6–7.5)	10.1% (5.6–17.6)	6.0% (2.3–14.4)	0.0% (0.0–10.2)	3.9% (1.1–13.2)	0.0% (0.0–18.4)	10.5% (2.9–31.4)	4.4% (3.0–6.4)
7 m–1y v	14 (24.1)	24 (22.0)	2 (1.6)	9 (12.3)	29 (26.4)	6 (15.8)	0	0	0	0	84 (15.2)
Positivity rates (95% CI*)	45.2% (29.2–62.2)	36.9% (26.2–49.1)	7.1% (2.0–22.6)	7.1% (3.8–13.0)	16.6% (11.8–22.8)	12.2% (5.7–24.2)	0.0% (0.0–16.1)	0.0% (0.0–7.9)	0.0% (0.0–32.4)	0.0% (0.0–16.8)	14.8% (12.1–18.0)
2y–5y (%)	12 (20.7)	9 (8.3)	6 (4.7)	13 (17.8)	16 (14.5)	1 (2.6)	1 (16.7)	6 (37.5)	0	4 (26.7)	68 (12.3)
Positivity rates (95% CI*)	30.0% (18.1–45.4)	20.9% (11.4–35.2)	16.7% (7.9–31.9)	16.5% (9.9–26.1)	12.3% (7.7–19.1)	4.0% (0.7–19.5)	10.0% (1.8–40.4)	13.6% (6.4–26.7)	0.0% (0.0–43.4)	16.7% (6.7–35.9)	15.6% (12.5–19.3)
6y–10y (%)	9 (15.5)	9 (8.3)	15 (11.8)	5 (6.8)	9 (8.2)	3 (7.9)	0	6 (37.5)	0	6 (40.0)	62 (11.2)
Positivity rates (95% CI*)	32.1% (17.9–50.7)	36.0% (20.2–55.5)	38.5% (24.9–54.1)	7.8% (3.4–17.0)	15.8% (8.5–27.4)	25.0% (8.9–53.2)	0.0% (0.0–39.0)	24.0% (11.5–43.4)	0.0% (0.0–65.8)	30.0% (14.5–51.9)	22.3% (17.8–27.6)
11y–20y (%)	6 (10.3)	13 (11.9)	15 (11.8)	12 (16.4)	18 (16.4)	6 (15.8)	0	0	0	0	70 (12.7)
Positivity rates (95% CI*)	27.3% (13.2–48.2)	18.3% (11.0–28.8)	20.0% (12.5–30.4)	10.0% (5.8–16.7)	11.3% (7.3–17.2)	7.0% (3.2–14.4)	0.0% (0.0–15.5)	0.0% (0.0–11.4)	0.0% (0.0–39.0)	0.0% (0.0–25.9)	11.6% (9.3–14.5)
21y–30y (%)	9 (15.5)	21 (19.3)	28 (22.0)	14 (19.2)	14 (12.7)	9 (23.7)	4 (66.7)	1 (6.3)	0	2 (13.3)	103 (18.6)
Positivity rates (95% CI*)	32.1% (17.9–50.7)	16.2% (10.8–23.4)	25.0% (17.9–33.8)	11.3% (6.8–18.1)	7.7% (4.7–12.6)	9.9% (5.3–17.7)	10.8% (4.3–24.7)	1.8% (0.3–9.6)	0.0% (0.0–29.9)	11.1% (3.1–32.8)	13.1% (10.9–15.7)
31y–40y (%) (%)	7 (12.1)	22 (20.2)	37 (29.1)	12 (16.4)	9 (8.2)	3 (7.9)	1 (16.7)	1 (6.3)	0	0	92 (16.6)
Positivity rates (95% CI*)	21.2% (10.7–37.8)	23.9% (16.4–33.6)	40.7% (31.1–50.9)	12.4% (7.2–20.4)	8.7% (4.7–15.8)	5.8% (2.0–15.6)	4.8% (0.8–22.7)	2.4% (0.4–12.6)	0.0% (0.0–39.0)	0.0% (0.0–24.2)	16.8% (13.9–20.1)
41y–50y (%)	1 (1.7)	4 (3.7)	13 (10.2)	5 (6.8)	3 (2.7)	2 (5.3)	0	0	0	1 (6.7)	29 (5.2)
Positivity rates (95% CI*)	16.7% (3.0–56.4)	30.8% (12.7–57.6)	44.8% (28.4–62.5)	13.9% (6.1–28.7)	10.0% (3.5–25.6)	7.7% (2.1–24.1)	0.0% (0.0–32.4)	0.0% (0.0–32.4)	0.0% (0.0–0.0)	20.0% (3.6–62.4)	18.0% (12.8–24.7)
51y–60y (%)	0	2 (1.8)	9 (7.1)	0	2 (1.8)	4 (10.5)	0	0	0	0	17 (3.1)
Positivity rates (95% CI*)	0.0% (0.0–39.0)	20.0% (5.7–51.0)	50.0% (29.0–71.0)	0.0% (0.0–22.8)	10.5% (2.9–31.4)	28.6% (11.7–54.6)	0.0% (0.0–49.0)	0.0% (0.0–35.4)	0.0% (0.0–79.3)	0.0% (0.0–0.0)	18.5% (11.9–27.6)
60y+ (%)	0	0	1 (0.8)	1 (1.4)	0	0	0	0	0	0	2 (0.4)
Positivity rates (95% CI*)	0.0% (0.0–79.3)	0.0% (0.0–56.1)	12.5% (2.2–47.1)	9.1% (1.6–37.7)	0.0% (0.0–16.1)	0.0% (0.0–49.0)	0.0% (0.0–79.3)	0.0% (0.0–43.4)	0.0% (0.0–0.0)	0.0% (0.0–0.0)	3.8% (1.0–12.8)

*95% CI: 95% Confidence Interval, calculated using the Wilson score method

In total, 132 cities across the entire state recorded at least one positive B19V case during the analyzed period. Most cases were concentrated in the state capital, Porto Alegre, and its metropolitan region, which includes 34 municipalities and concentrates 38% of the state’s population, where 241 of the 553 positive cases were recorded (Fig. 1b). Of these 553 ELISA-positive samples, 160 were subsequently tested by real-time PCR, all of which were positive for B19V. Real-time PCR of these 160 samples showed a range of viral loads, with Ct values spanning 9.0 to 40.0. The overall median Ct was 34.0 (mean = 32.26; SD = 6.61) (Fig. 1c). Stratification by age group showed significant variation in estimated viral loads (Kruskal-Wallis, *p* < 0.0001; Supplementary Fig. 2). Adults aged 26–35 years (median Ct = 29.5) and 36–45 years (median Ct = 30.0) exhibited significantly lower Ct values, similar to women of childbearing age (15–49 years), who presented a median Ct of 31.0. B19V infections increased during winter months (July in the Southern Hemisphere), with a more pronounced peak in September. The year with the highest number of detected cases was 2017 (*n* = 128), during which January showed the highest monthly count (*n* = 34), indicating an atypical early-year peak. Despite these fluctuations, B19V circulated throughout all months of the year (Fig. 1d)

### Genomic characteristics and phylodynamics of B19V in Rio Grande do Sul, Brazil

Overall, the WGS-B19V primer scheme showed robust compatibility with the virus’s known global genomic diversity. Across the 503 sequences, the primers showed high homology: 71.8% of primer-genome comparisons matched perfectly (zero mismatches), and 18.5% had a single-nucleotide mismatch. The detailed overall mismatch count for each primer is provided in https://github.com/salvatolab/B19V-genomic-epidemiology-Rio-Grande-do-Sul. We observed that these minor divergences were predominantly localized to the 5′ regions of the primers. Across all primer–genome comparisons, 95.7% showed no mismatches within the last 3 nucleotides of the primers, with only a minimal proportion presenting one or more mismatches in this critical region. This high level of conservation at the 3′ ends, which dictate proper primer annealing and polymerase extension, confirms that the overall primer mismatches do not translate into meaningful genomic dropout, supporting the robustness and broad applicability of the primer scheme for B19V whole-genome amplification.

Of the 55 B19V genomes sequenced in this study (five by metagenomic and 50 using our amplicon-based protocol), 47 were included in the final analysis. The included genomes showed median sequencing depths ranging from 139× to 22,643× and genome coverage breadths ranging from 70% to 100%. In contrast, the eight excluded samples had insufficient mean sequencing depth and genome coverage below 70%, making them unsuitable for reliable phylogenetic inference. Sequencing of the negative control yielded a mean coverage of 0.0491, as expected, providing no evidence of contamination. Quality metrics for all sequenced samples are available at https://github.com/salvatolab/B19V-genomic-epidemiology-Rio-Grande-do-Sul.

Our B19V dataset comprised 498 genomes: 47 newly generated sequences, 446 curated publicly available genomes, the NC_000883.2 reference genome, and four genotype reference strains. Phylogenetic analysis recovered the three known B19V genotypes, comprising 443 genomes in genotype 1, 32 in genotype 2, and 23 in genotype 3, with ultrafast bootstrap support values of 100, 99, and 100, respectively. All newly generated genomes clustered within genotype 1 (Supplementary Fig. 1). Of the 443 genotype 1 genomes, 423 had interpretable collection dates and were retained for time-resolved phylogenetic analysis: 47 from this study and 376 publicly available genomes, including 34 from other Brazilian states [16, 17].

**Fig. 2:**
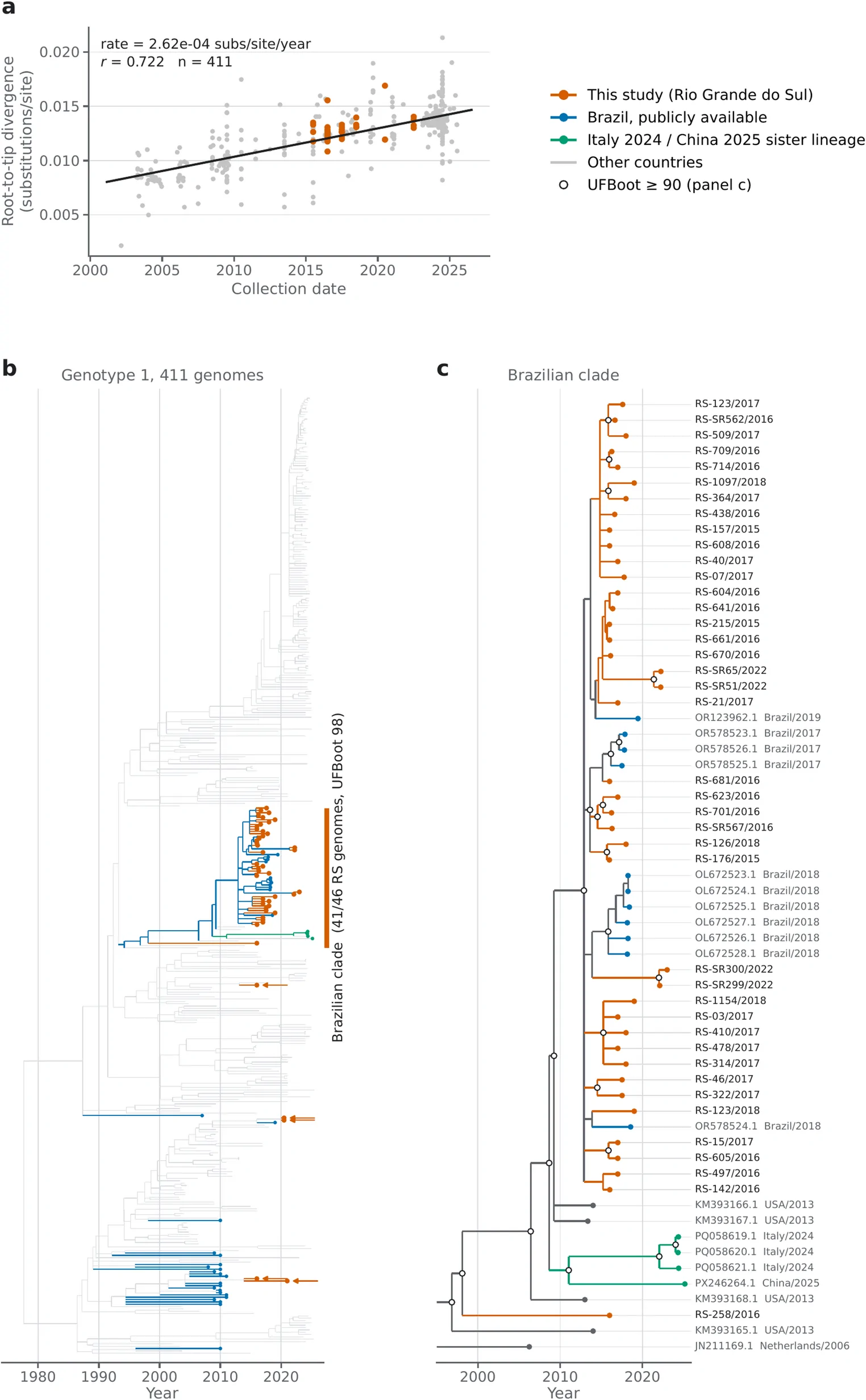
Dated phylogeny of parvovirus B19 genotype 1 and structure of the Rio Grande do Sul genomes (RS). **(a)** Root-to-tip divergence against collection date for the 411 genotype 1 genomes, with the genomes generated in this study in orange. **(b)** Time-calibrated tree of the same 411 genomes. Tips generated in this study are in orange, publicly available Brazilian genomes in blue, the Italian and Chinese sister lineage in green, and all other countries in grey. The bar on the right delimits the Brazilian clade; arrowheads mark the six genomes from Rio Grande do Sul that fall outside it. **(c)** The Brazilian clade in detail, with tip labels. Open circles mark nodes with ultrafast bootstrap of at least 90

Eleven publicly available genomes and one genome generated in this study deviated from the molecular clock regression by more than four interquartile ranges and were excluded, leaving 411 genomes. Root-to-tip divergence increased with collection date at a rate of 2.6 × 10⁻⁴ substitutions per site per year (95% CI 2.4–2.9 × 10⁻⁴; correlation coefficient = 0.72, *p* < 0.001, *n* = 411; Fig. 2a). The time-calibrated phylogeny (Fig. 2b) estimated the most recent common ancestor of genotype 1 to date to late 1977 (95% CI: 1973.8–1982.6), with an estimated molecular clock rate of 2.0 × 10⁻⁴ (± 2.2 × 10⁻⁵) substitutions per site per year.

Forty-one of the 46 genomes from Rio Grande do Sul included in the time-resolved analysis clustered within a single clade (ultrafast bootstrap support = 98), whose most recent common ancestor was estimated to date to 1994.2 (95% CI: 1992.8–1997.0). This clade also included 11 genomes from other Brazilian states and a few genomes from the United States, Italy, the Netherlands, and China (Fig. 2b and c). Within this clade, a group of 51 genomes shared a most recent common ancestor dating to 2012.9 (95% CI: 2011.0–2014.7; ultrafast bootstrap support = 90). This group consisted exclusively of Brazilian genomes: 40 from Rio Grande do Sul and 11 from other states. The sister lineage to this Brazilian clade comprised three Italian genomes sampled in 2024 and one Chinese genome sampled in 2025 (ultrafast bootstrap support = 99). The most recent common ancestor shared by the Brazilian clade and its sister lineage was estimated to date to 2008.7 (95% CI: 2005.9–2010.2; ultrafast bootstrap support = 99). Genomes from Rio Grande do Sul were interspersed with sequences from other Brazilian states throughout the clade, rather than forming a single state-specific cluster (Fig. 2c). One small group included a genome sampled in 2016 and two genomes sampled in 2022. The five remaining genomes from Rio Grande do Sul fell outside the Brazilian clade, within the other basal lineage of genotype 1. These comprised two pairs clustering with sequences from other Brazilian states and one genome that was sister to a sequence from Argentina.

## Discussion

Our study provides a comprehensive epidemiological and genomic overview of B19V circulation in Rio Grande do Sul, southern Brazil, over a ten-year period, indirectly inferred through surveillance of exanthematous diseases. B19V infections occurred across all age groups but were more frequently detected in women and adults aged 21–40 years, consistent with previously reported findings in Brazil [16, 18] and other countries [3, 11]. Notably, adults aged 26–45 years also had significantly lower Ct values in our study, consistent with studies reporting higher B19V viral loads among younger adults. However, these findings should be interpreted in the context of our passive surveillance system, where testing is more likely to occur in adults with clinically significant presentations rather than through systematic population screening, potentially increasing the representation of more severe cases with higher viral loads [19].

We observed sustained year-round B19V circulation with winter seasonal peaks, highlighting the importance of including B19V in the differential diagnosis of febrile exanthematous illnesses. As B19V is not a notifiable disease in Brazil, its epidemiological burden remains poorly characterized, particularly outside the existing surveillance framework for exanthematous diseases. Our detection of 553 B19V-positive cases, compared with the scarcity of previously published data from Rio Grande do Sul, highlights a major surveillance gap. Given the risk of severe complications in pregnant women, including hydrops fetalis and fetal death, as well as in immunocompromised individuals and patients with hematological disorders, expanding laboratory surveillance and improving access to B19V diagnosis are important considerations for public health.

Phylogenetic analysis showed that all sequenced genomes from Rio Grande do Sul belonged to genotype 1. This pattern mirrors findings from other Brazilian regions, where genotype 1 consistently predominates, suggesting long-standing endemic circulation with limited introduction of external lineages. Globally, different B19V genotypes exhibit distinct geographic distributions, and the apparent genotypic homogeneity observed here hides structure below the genotype level. Most of the genomes from Rio Grande do Sul, 41 of the 46 in the dated phylogeny, belong to a clade that also holds sequences from other Brazilian states and dates to the mid-1990s, and the exclusively Brazilian core of that clade traces back to the early 2010s. Its internal structure reflects a virus transmitted locally and repeatedly: genomes from Rio Grande do Sul are interleaved with sequences from other states rather than descending from them, and one small group links samples six years apart. The five genomes outside this clade sit next to sequences from other Brazilian states, and from Argentina, so even the diversity outside the Brazilian clade points to circulation within South America rather than recent introductions from other continents. The sister lineage of the Brazilian clade extends beyond Brazil: three Italian genomes from 2024, sampled during the European B19V resurgence [20, 21], and one Chinese genome from 2025 share an ancestor with the Brazilian clade around 2009. The current genotype classification system, which largely relies on partial NS1 sequences [21], does not adequately capture this internal diversification. Our findings add to growing evidence that whole-genome-based phylogenetic approaches or intragenotype-level classifications may better represent B19V evolutionary diversity, with potential epidemiological and clinical relevance.

B19V circulated in Rio Grande do Sul throughout the ten years covered here, and the genomic data show sustained local transmission rather than successive isolated introductions. Detection, however, was uneven, with positivity among tested samples ranging from 28.2% in 2015 to 3.7% in 2021 (*p* < 0.001), and part of that unevenness reflects underreporting during outbreaks of other infectious diseases, when healthcare systems were overwhelmed and diagnostic priorities shifted. In contrast to Europe, where the B19V upsurgence in 2024 has been associated with a substantial immunity gap among younger populations and atypical, sustained transmission following the COVID-19 pandemic, Rio Grande do Sul showed no comparable rebound. Several factors may account for this difference [3]. First, the local immunity gap may have been smaller, suggesting that pandemic restrictions did not completely interrupt B19V circulation and natural immunization. Second, B19V appears to have retained its typical seasonal pattern, without the prolonged off-season transmission that may have contributed to the European outbreaks.

Underdiagnosis may also have contributed to the absence of an apparent resurgence. Dengue has recently become endemic in Rio Grande do Sul, a state that until recently reported predominantly imported cases, with incidence increasing substantially from 2020 onwards [22]. This rapid expansion of dengue in Rio Grande do Sul during the latter years of our study period likely masked B19V infections, as both viruses share overlapping clinical presentations, including fever, rash, and myalgia, making differential diagnosis particularly challenging in outpatient settings. Supporting this hypothesis, Di Paola et al. [23] detected B19V viremia in 63% of dengue-suspected cases, highlighting how frequently B19V infections are masked by overlapping clinical syndromes. Garcia and Leon [18] similarly reported that B19V outbreaks in Brazil often go unnoticed during arbovirus epidemics. However, our study did not directly test for co-infections or systematically evaluate arbovirus-suspected cohorts. Therefore, the “dengue-masking” scenario remains a hypothesis rather than a conclusion established by our data.

Nevertheless, the detection of B19V throughout the study period, together with its overlapping clinical presentation with arboviral infections, highlights the need to strengthen differential diagnosis in regions where these pathogens co-circulate. Our findings support including B19V in diagnostic panels for febrile exanthematous illnesses, particularly during periods of increased arbovirus circulation, when clinically similar infections may be overlooked. Special attention should be given to pregnant women presenting with febrile rash illness, for whom B19V serological testing should be considered regardless of arbovirus test results, given the potential for coinfection and the risk of severe fetal complications. Future studies incorporating systematic B19V testing in both arbovirus-positive and arbovirus-negative cohorts are needed to better characterize viral co-circulation, assess the extent of diagnostic misattribution, and inform integrated surveillance strategies.

Our study estimated a clock rate of 2.0 × 10⁻⁴ substitutions per site per year for B19V (± 2.2 × 10⁻⁵), with the independent root-to-tip regression giving 2.6 × 10⁻⁴. This rate is high for a DNA virus and approaches the range observed in RNA viruses. Previous studies have also highlighted unexpectedly high substitution rates for B19V. For instance, Shackelton [24] reported a substitution rate on the order of 10⁻⁴ substitutions/site/year, and Stamenković [25] estimated a rate of 1.03 (0.6–1.27) × 10⁻⁴ substitutions/site/year, supporting the idea that B19V evolves faster than initially predicted for a single-stranded DNA virus. Our estimate falls in the same order of magnitude as both.

This apparent contrast between the high mutation rate observed in molecular clock analyses and the relatively conserved phylogenetic structure over time may reflect a balance between rapid nucleotide-level mutation and strong purifying selection, which constrains functional changes and maintains overall genomic stability [26, 27]. Moreover, using host cellular DNA polymerase for replication likely contributes to a lower intrinsic replication error rate than RNA viruses, yet overall genetic variability remains considerable because the virus circulates long-term in distinct populations [9, 28]. Taken together, these findings reinforce the notion that B19V displays evolutionary flexibility and underline the importance of ongoing molecular surveillance to better understand the mechanisms driving its diversity and potential implications for pathogenicity and immune evasion.

Despite the importance of our findings, this study has important limitations that should be acknowledged. The genomic dataset, while representing the largest collection of near-complete B19V genomes from Southern Brazil to date, remains relatively small, which may limit the resolution of phylodynamic inferences such as tMRCA estimates and ancestral state reconstructions. Furthermore, as B19V infection is not a notifiable disease, testing is performed only as a complementary diagnosis for suspected measles or rubella. This introduces a selection bias, as the tested population is limited to individuals with febrile exanthematous illness who tested negative for measles, rather than representing the general population or clinically suspected B19V cases. In addition, B19V PCR testing was restricted to samples collected within five days of symptom onset, when viremia is more likely to be detectable. This strategy may have increased the likelihood of identifying active B19V infections but also limits the findings primarily to the viremic phase of infection and may underestimate infections detected later in the clinical course. Year-to-year comparisons of positivity therefore describe the samples tested, not population incidence, since both the number and profile of samples reaching LACEN-RS changed over the period. In addition, a substantial proportion of individuals presenting with suspected arboviral infections are not captured by the current testing algorithm, further constraining the identification of B19V infections within this population. The publicly available context is also geographically uneven: France, the Netherlands and China together account for more than half of the genotype 1 genomes with collection dates, so the position of the Brazilian clade relative to other regions rests on sparse sampling outside Europe and East Asia.

## Conclusions

Our study provides a comprehensive overview of the epidemiological patterns, genomic diversity, and evolutionary dynamics of B19V circulating in Rio Grande do Sul, Southern Brazil, over a ten-year period. The sustained circulation of genotype 1, combined with clear winter seasonal peaks and the identification of a well-supported Brazilian clade shared with other states, alongside genomes grouping with lineages sampled elsewhere in South America, highlights the endemic nature and evolutionary complexity of B19V in the region. Our findings underscore the value of whole-genome sequencing for improving phylogenetic resolution beyond current genotype-level classifications and emphasize the need to strengthen B19V laboratory surveillance in Brazil. Integrating B19V testing into diagnostic algorithms for febrile exanthematous illnesses, particularly during periods of increased arbovirus circulation, could improve case recognition and provide a more accurate understanding of its epidemiological burden. Particular attention should be given to pregnant women and other vulnerable populations, for whom timely diagnosis is important because of the potential for severe complications. These measures, combined with expanded genomic surveillance, could improve our understanding of B19V circulation and support more informed public health responses.

## Supplementary Information

Below is the link to the electronic supplementary material.

Supplementary Material 1

## Data Availability

All genome sequences generated in this study have been deposited in GenBank under accession numbers PZ316512-PZ316558. All supplementary material, scripts and supporting information are additionally available at https://github.com/salvatolab/B19V-genomic-epidemiology-Rio-Grande-do-Sul.
