# Supplementary material for: Genomic epidemiology of human parvovirus B19 in Southern Brazil, 2015–2024
Source: Mol Biol Rep. 2026 Oct 30;53(1):1867. doi: 10.1007/s11033-026-13080-3 (PMC13722631; doi:10.1007/s11033-026-13080-3)
Supplement: Supplementary file 1 [file 11033_2026_13080_MOESM1_ESM.docx]

**Supplementary Materials**

***Supplementary Table 1****. Primer scheme designed for amplification of the complete sequence of parvovirus B19. Primer scheme was designed by varVAMP v.1.1.2* (https://github.com/jonas-fuchs/varVAMP).

| Primer name | Sequence 5’-3’ | Melting temperatures (Tm)* | Amplicon size (bp) |
| --- | --- | --- | --- |
| WGS_B19V_0_ RIGHT | GCAAGCTCCCCTTCTAAAAGTAGC | 57.4 °C | 970 |
| WGS_B19V_0_LEFT | AGCTTGCTTGGGGTTGCCT | 55.4 °C | 970 |
| WGS_B19V_1_RIGHT | TCATCCCAGACCACCAAGCTTT | 54.8 °C | 845 |
| WGS_B19V_1_ LEFT | AACCCCAGAAACCTHACAVTGT | 51.1 – 54.8 °C | 845 |
| WGS_B19V_2_RIGHT | TTCACCACCACTGCTGCTGATA | 54.8 °C | 635 |
| WGS_B19V_2_LEFT | TATTGGTGGGGCAGCATGTGT | 54.4 °C | 635 |
| WGS_B19V_3_RIGHT | TACGCTAACTTGCCCAGGCTT | 56.3 °C | 974 |
| WGS_B19V_3_LEFT | TGCACAAAGCTGGGACCACTA | 54.3 °C | 974 |
| WGS_B19V_4_ RIGHT | GCCTCCTTTCCACTGGCATTG | 58.2 °C | 742 |
| WGS_B19V_4_LEFT | CCCTAGAAAACCCATCCTCTYTGT | 55.6 – 57.4 °C | 742 |
| WGS_B19V_5_RIGHT | TTTGGGGCTGAATTGCATGGTC | 56.7 °C | 776 |
| WGS_B19V_5_LEFT | GGCCACTTTTAGTCCYAAYTCTG | 53.5 – 57.0 °C | 776 |
| WGS_B19V_6_RIGHT | TGGTACTGGTGGGCGTTTAGTT | 54.8 °C | 995 |
| WGS_B19V_6_LEFT | AGAGGGCTGCAGTCAACACTT | 54.3 °C | 995 |
| WGS_B19V_7_RIGHT | GCMCAGAGCCAACCCTAAT | 51.1 – 53.2 °C | 615 |
| WGS_B19V_7_LEFT | ACCCYACAGCTACAGATGCAA | 54.3 – 56.3 °C | 615 |

* For degenerate primers, Tm is expressed as a range (min–max) covering all sequence combinations.

***Supplementary Figure 1.*** *Maximum likelihood phylogenetic tree of 498 parvovirus B19 genomes showing the three genotypes. Branch support is ultrafast bootstrap, shown at the genotype nodes. Genotypes are highlighted with coloured backgrounds. Tips generated in this study are shown in orange, publicly available genomes in grey, and genotype reference strains are marked with diamonds. The two basal branches carry break marks and are drawn shortened, so that the internal structure of each genotype stays legible.*

***Supplementary Figure 2.*** *Distribution of Parvovirus B19 Ct values across different age groups. Boxplots represent the median and interquartile range, with individual patient samples superimposed as scattered points (blue for males, red for females). We evaluated overall variance among age groups using the Kruskal-Wallis test (p < 0.0001). We performed pairwise comparisons against the pediatric reference baseline (0–12 years) using the Wilcoxon rank-sum test. Statistical significance is denoted by asterisks: * p ≤ 0.05; *** p ≤ 0.001; **** p ≤ 0.0001 (ns: non-significant).*
